# Synthesis of the Nakanishi Ring-Locked Retinoid

**DOI:** 10.1155/2011/826792

**Published:** 2011-10-05

**Authors:** Jamie B. Côté, Tan D. Quach, Andrey P. Demenev, David S. Garvey, Judd M. Berman

**Affiliations:** ^1^Dalton Medicinal Chemistry Inc., 349 Wildcat Road, Toronto, ON, Canada M3J 2S3; ^2^Dalton Pharma Services, 349 Wildcat Road, Toronto, ON, Canada M3J 2S3; ^3^Bikam Pharmaceuticals Inc., c/o Fidelity Biosciences, 1 Main Street, 13th Floor, Cambridge, MA 02142, USA

## Abstract

An optimized synthetic route to prepare ring-locked retinoid **1a** has been developed. We fully describe a purification protocol that provides isomerically pure **1a** in support of on-going proof of concept studies for the development of therapeutic agents to treat human ADRP. Additionally, we have found that isomerically pure **1a** can be stored in amber vials under argon at −20°C for use over time (up to six months) without degradation. Thus, enabling **1a** to be an accessible and valuable biological tool.

Previous studies using P23H mutant opsin as an *in vitro* model of human Autosomal Dominant Retinitis Pigmentosa (ADRP) found that ring-locked retinoid **1a** (Figure 1, carbon numbering shown based on 11-*cis*-retinal) was able to act as a pharmacological chaperone [1–3]. Compound **1a** induced the mutant protein to fold properly and undergo normal cellular transport and degradation suggesting **1a** could be a potential therapeutic agent for the prevention of ADRP [1–3]. This ring-locked analog of 11-*cis*-retinal has also been used extensively to elucidate the mechanism of photoactivation of rhodopsin [4–6].

We required a reliable synthesis of isomerically pure compound **1a**. Its synthesis, along with the spectral data, has been reported twice in the literature [1, 7], in addition to the syntheses of a number of related ring-locked retinoids [4–6, 8]. The original synthetic route reported by Nakanishi resulted in the preparation of mixtures of multiple geometric isomers of **1** (*E/Z*-isomers among C7-C8, C9-C10, and C13-C14) and did not describe specific HPLC conditions for resolution of the final isomerically pure products. Our synthetic strategy differs from the two reported syntheses in two fundamental ways (discussed below) enabling ready access to **1a**. Two key bond-forming events distinguish this synthesis: the C7-C8 double bond was originally established via a Julia olefination, while the connection between C13 and C14 was accomplished via a Petersen olefination. In each case, both *E-* and *Z*-isomers of the desired products were reported to have formed [7]. In contrast, our use of a Wittig reagent in the formation of the C7-C8 double bond afforded the *E*-isomer as the sole product in the former olefination; and in the case of the latter, the requisite Petersen reagent, a silylated acetaldehyde *tert*-butylamine, was not readily accessible in our hands, and an alternate methodology was instead utilized [7, 9]. A Horner-Emmons-Wadsworth strategy (utilizing diethyl cyanomethylphosphonate) was found to be an advantageous replacement for the penultimate formation of the C13-C14 double bond in two steps (as opposed to three as previously reported) [1]. In particular, using the nitrile Horner-Emmons-Wadsworth (Scheme 2) reagent obviated the need for a final manganese dioxide oxidation which resulted in a more efficient and reliable synthesis endgame for the preparation of **1a**. The key changes we have introduced to the synthetic route as described by Noorwez et al. [1] allowed us to increase the yields and geometric purity of key intermediates (**7**, **11**). Herein, we report the full details of the synthesis, purification, characterization, and stability of isomerically pure **1a**.

Starting from commercially available cycloheptenone **2**, the allylic acetate **4** was prepared via a two-step sequence of radical bromination with NBS and 1,1′-azobis(cyclohexanecarbonitrile) (ACHN) followed by displacement of the allylic bromide **3** with potassium acetate under phase transfer catalyst conditions with tetrabutylammonium bromide (TBAB, Scheme 1). The ketone underwent Horner-Emmons-Wadsworth olefination with diethyl(1-cyanoethyl)phosphonate to provide a 50% yield of **5** and **6** with an *E *:* Z* ratio of 2 : 1. The isomers were separated by column chromatography, and the geometry of the double bond was established by NOE (Figure 2). The allylic acetate **5** was hydrolyzed with potassium carbonate in methanol to furnish the allylic alcohol **7** in excellent yield and purity. The alcohol was protected as the TBS ether **8** in the presence of imidazole and DMAP, and then the nitrile was reduced with DIBAL-H to provide aldehyde **9** in 77% yield over two steps. The aldehyde **9** was carried forward without purification.

Importantly, the double-bond geometry (between C9 and C10) remained fixed during this three-step reaction sequence. The *trans* geometry was confirmed by NOE experiments (key interactions are shown in Figure 2).

The Wittig olefination reagent, *β*-cyclogeranyl triphenylphosphonium bromide, was prepared following established methodology [10, 11]. Reaction conditions using *n*-BuLi as the base resulted only in decomposition. However, the desired tetraene **11** was obtained when potassium *tert*-butoxide was employed as the base in the presence of a catalytic amount of 18-crown-6 [12]. In addition, the newly established C7-C8 double bond was solely of the *trans* geometry. The *cis* isomer of **11** was not obtained or detected due to judicious choice of conditions, optimization of the reaction time/temperature, and the inherent instability of the C7/C8 *cis* isomer due to the unfavorable sterics between the C5/C9 methyl groups. Interestingly, compound **11** was found to be quite stable when stored at 4°C in the dark under argon (no decomposition or isomerization detected after 18 months). The TBS protecting group was cleaved using TBAF to provide the corresponding allylic alcohol. In contrast to **11**, we found the allylic alcohol readily decomposed under normal atmospheric conditions; thus, it was more convenient to oxidize it directly using MnO_2_ to provide ketone **12** which, in our hands, was more stable to manipulation. The Horner-Emmons-Wadsworth reaction on **12** with diethyl cyanomethylphosphonate provided the corresponding nitrile in excellent yield as a 1 : 1 mixture of *E-* and *Z-*isomers which, as reported on similar analogues, were not separable by HPLC [8]. Thus, reduction of the nitrile was achieved by treatment with DIBAL-H to the intermediate imine, and silica gel-mediated hydrolysis furnished the aldehydes **1a** and **1b** in high yield. The observed ratio of C13-C14 double-bond isomers and reaction yield is related to the conditions of the final step of synthesis. Hence, if hydrolysis of the intermediate imine from the conversion of **12** to **1** on silica gel is left longer than 30 minutes at room temperature, more isomerization and decomposition takes place (resulting in the appearance of three other peaks in the HPLC trace), and the yield of **1a/b** decreases. Under the conditions reported herein, the combined percentage of the other impurities in addition to the C13-C14 *E*/*Z*-isomers was minimal (4% AUC at 350 nm). Both the C13-C14 *E/Z*-isomers of **1** were readily separated by normal phase HPLC in a dark room (see Experimental). The isomers were identified by comparison of key chemical shifts in the ^1^H NMR spectra to literature data (Table 1) [1, 7]. 

It was observed that the isomerization of the C13-C14 double bond of **1** occurs readily in solution under normal atmospheric conditions regardless of exposure to light. Hence, a crude sample isolated from the reaction as a 1 : 1 mixture of **1a** and **1b** would isomerize between the two compounds.

To probe the isomers' stability to light and storage conditions, 2 mg of each purified isomer was dissolved in hexane (2 mL) in a clear glass vial. Half of this solution was transferred into an amber glass vial. Each of the vials was flushed with argon and left to stand at room temperature in ambient light. After one hour, aliquots were removed from each vial and analyzed by HPLC. The samples in the amber vials remained unchanged, whereas the samples in the clear vials had isomerized to 13% (AUC at 350 nm). The vials were flushed with argon again and left overnight at room temperature. Again, aliquots from each vial were analyzed by HPLC, the samples in the amber vials remained unchanged, but the samples in the clear vials had isomerized further to 31% (Figure 3).

Importantly, in a separate experiment, a purified sample of **1a** was stored neat at −20°C in an amber vial flushed with argon. It was found that the sample remained unchanged from isomerization or decomposition (via HPLC monitoring) after six months.

In conclusion, we have developed a synthetic route to prepare the ring-locked retinoid **1a** and described a reproducible purification protocol by normal phase HPLC to isolate the isomerically pure C13-C14 *Z*-isomer of **1**. Finally, we have found that under proper conditions **1** can be stored for extended periods of time (up to six months) without degradation. 

^1^H, ^13^C spectra were recorded on a Bruker ARX 300, 400, or 600 MHz spectrometers. Low-resolution mass spectra (MS) were obtained on a Thermo Finnigan LCQ Classic or LCQ Duo spectrometer. HPLC purifications were conducted on a Waters Prep LC 4000 equipped with a Waters plus 717 autosampler and a Waters 2487 dual *λ* Absorbance Detector.

All commercial reagents were used as received, unless otherwise noted. All reactions, work-up, and purifications for compounds **1**, **9–12** were performed in a dark room utilizing a red spectrum light source. Final compounds and isolated key intermediates were stored in amber glass vials at −20°C under argon. Analytical thin-layer chromatography (TLC) was performed on glass-backed TLC plates (silica gel 60 F_254_ from Silicycle), visualized with a UV lamp (254 nm), and stained with a basic solution of KMnO_4_. Flash column chromatography was conducted on an ISCO Companion automated chromatographer using generic silica gel cartridges from Silicycle.

**4-Oxo-cyclohept-2-en-1-yl Acetate (4) (See [12])**

A solution of cycloheptenone (**2**, 80% tech. grade, 8.00 g, 73.0 mmol), NBS (17.5 g, 98.0 mmol), and ACHN (180 mg, 0.730 mmol) in benzene (150 mL) was combined at r.t. under argon then heated at reflux for 3 h. The reaction mixture was cooled to room temperature, then cooled to 0°C in an ice bath, and diluted with hexanes (200 mL). The succinimide precipitate was removed by vacuum filtration, and the filtrate was concentrated *in vacuo* to provide **3** as a dark brown oil. The crude bromide **3** was dissolved in THF (75 mL) at room temperature. To this solution was added water (50 mL), potassium acetate (28.0 g, 285 mmol), and tetrabutylammonium bromide (230 mg, 0.730 mmol). The biphasic mixture was stirred vigorously at r.t. for 4 days under argon. The reaction mixture was then transferred to a separatory funnel and extracted with ether (3 × 100 mL). The combined organic phases were washed with saturated NaHCO_3_ (2 × 150 mL), brine (150 mL), dried over MgSO_4_, filtered, and concentrated *in vacuo.* The product was purified by column chromatography (330 g silica gel cartridge, gradient elution 0–20% EtOAc-hexanes), yield of **4**, 2.0 g (12 mmol, 17%), and recovered **3**, 4.6 g (24 mmol, 34%).

*R_f_* = 0.21 (EtOAc-hexanes, 1 : 8).

^1^H NMR (600 MHz, CDCl_3_): *δ* = 6.42 (dd, 1H, *J *= 12.5, 3.5 Hz, H-2), 6.00 (dd, 1H, *J *= 12.5, 1.5 Hz, H-3), 5.56 (m, 1H, H-1), 2.66–2.56 (m, 2H, H-5), 2.22–2.15 (m, 1H, H-7), 2.10–2.06 (s, 3H, O_2_CCH_3_), 1.90–1.81 (m, 3H, H-6, H-7).

^13^C NMR (150 MHz, CDCl_3_): *δ* = 18.0 (C6), 21.0 (O_2_CCH_3_), 31.6 (C7), 42.8 (C5), 71.9 (C1), 131.3 (C3), 144.3 (C2), 169.9 (O_2_CCH_3_), 202.3 (C4).

MS (APCI): *m/z* [M + H]^+^ = 169.1.

**4-Bromocyclohept-2-enone (3) (See [13])**

*R_f_* = 0.34 (EtOAc-hexanes, 1 : 8).

^1^H NMR (600 MHz, CDCl_3_): *δ* = 6.50 (dd, 1H, *J *= 12.5, 5.5 Hz, H-3), 5.90 (d, 1H, *J *= 12.5 Hz, H-2), 4.99 (dd, 1H, *J *= 10.0, 5.5 Hz, H-4), 2.81 (ddd, 1H, *J *= 17.0, 7.5, 4.5 Hz, H-7), 2.68–2.58 (m, 1H, H-7), 2.46–2.28 (m, 2H, H-5), 2.19–2.08 (m, 1H, H-6), 1.91–1.78 (m, 1H, H-6).

^13^C NMR (150 MHz, CDCl_3_): *δ* = 19.2 (C6), 36.4 (C5), 43.8 (C7), 49.9, (C4), 130.6 (C2), 141.3 (C3), 202.3 (C1).

MS (APCI): *m/z* [M + H]^+^ = 189.1, 191.1.

**(*E*)-4-(1-Cyanoethylidene)cyclohept-2-en-1-yl Acetate (5) (See [12])**

Into a dried flask fitted with an addition funnel was placed NaH (60% dispersion in oil, 200 mg, 84.0 mmol) and THF (140 mL). The slurry was cooled in an ice bath, and the addition funnel was charged with a solution of diethyl(1-cyanoethyl)phosphonate (1.06 mL, 6.00 mmol) in THF (45 mL). The phosphonate solution was added slowly over 5 minutes to the NaH slurry at 0°C. The reaction mixture was then warmed to r.t. over 30 minutes. A solution of **4** (900 mg, 5.40 mmol) in THF (30 mL) was added via addition funnel to the phosphonate solution over 5 minutes. The reaction mixture was then stirred at r.t. for 18 h. The reaction was quenched by the addition of water (200 mL) and then extracted with ether (3 × 150 mL). The combined organic phases were dried over MgSO_4_, filtered, and concentrated *in vacuo.* The *E-* and *Z*-isomers were separated by column chromatography (120 g silica gel cartridge, isocratic elution 10% EtOAc-hexanes); yield of **5**, 0.36 g (1.8 mmol, 33%) and **6**, 0.23 g (1.1 mmol, 17%).

*R_f_* = 0.43 (EtOAc-hexanes, 1 : 8).

^1^H NMR (600 MHz, CDCl_3_): *δ* = 6.36 (d, 1H, *J *= 12.0 Hz, H-5), 5.96 (dd, 1H, *J *= 12.0, 2.0 Hz, H-4), 5.50 (m, 1H, H-6), 2.85 (td, 1H, *J *= 14.0, 5.0 Hz, H-9), 2.60–2.53 (m, 1H, H-9), 2.09 (s, 3H, O_2_CH_3_), 2.03–1.97 (m, 1H, H-7), 1.95 (s, 3H, C2-CH_3_), 1.91–1.75 (m, 3H, H-7, H-8).

^13^C NMR (150 MHz, CDCl_3_): *δ* = 16.3 (C2-CH_3_), 21.1 (O_2_CCH_3_), 21.9 (C8), 32.0 (C7), 34.2 (C9), 72.0 (C6), 106.4 (C3), 119.6 (C2), 127.1 (C5), 138.3 (C4), 151.9 (C1), 170.1 (O_2_CCH_3_).

LRMS (EI): *m/z* (%) = 205.1 (40), 163.1 (100).

**(*Z*)-4-(1-Cyanoethylidene)cyclohept-2-en-1-yl Acetate (6)**

*R_f_* = 0.33 (hexanes-EtOAc, 8 : 1).

^1^H NMR (600 MHz, CDCl_3_): *δ* = 6.64 (d, 1H, *J *= 12.0 Hz, H-5), 5.90 (dd, 1H, *J *= 12.0, 2.0 Hz, H-4), 5.51 (m, 1H, H-6), 2.62 (td, 1H, *J *= 15.0, 5.0 Hz, H-9), 2.40–2.31 (m, 1H, H-9), 2.07 (s, 3H, O_2_CH_3_), 2.04–1.98 (m, 1H, H-7), 1.96 (s, 3H, C2-CH_3_), 1.86–1.67 (m, 3H, H-7, H-8).

^13^C NMR (150 MHz, CDCl_3_): *δ* = 16.1 (C2CH_3_), 20.7 (C8), 21.1 (O_2_CCH_3_), 29.8 (C9), 31.9 (C7), 71.7 (C6), 106.7 (C3), 119.0 (C2), 130.0 (C5), 136.7 (C4), 152.7 (C1), 170.1 (O_2_CCH_3_).

LRMS (EI): *m/z* (%) = 205.1 (15), 163.1 (100).

**(*E*)-2-(4-Hydroxycyclohept-2-en-1-ylidene)propanenitrile (7) (See [12])**

To a solution of **5** (320 mg, 1.60 mmol) in methanol (10 mL) at 0°C was added K_2_CO_3_ (110 mg, 0.780 mmol). The reaction mixture was stirred for 15 minutes at 0°C, then warmed to r.t., and stirred for 1 h. The reaction mixture was poured into water (25 mL) and extracted with EtOAc (3 × 20 mL). The combined organic phases were then washed with brine (15 mL), dried over MgSO_4_, filtered, and concentrated *in vacuo.* The product was purified by column chromatography (40 g silica gel cartridge, gradient elution 5–40% EtOAc-hexanes); yield of **7**, 0.25 g (1.6 mmol, 99%).

*R_f_* = 0.11 (EtOAc-hexanes, 1 : 6).

^1^H NMR (300 MHz, CDCl_3_): *δ* = 6.26 (d, 1H, *J *= 12.0 Hz, H-5), 6.07 (d, 1H, *J *= 12.0 Hz, H-4), 4.48 (m, 1H, H-6), 2.79 (td, 1H, *J *= 10.0, 5.0 Hz, H-9), 2.47 (m, 2H, H-9, OH), 2.00 (m, 1H, H-7), 1.92 (s, 3H, C2-CH_3_), 1.87–1.61 (m, 3H, H-7, H-8).

^13^C NMR (75 MHz, CDCl_3_): *δ* = 16.2 (C2CH_3_), 22.1 (C8), 34.2 (C9), 35.6 (C7), 69.9 (C6), 105.4 (C3), 119.8 (C2), 125.4 (C5), 143.0 (C4), 152.7 (C1).

MS (APCI): *m/z* [M + H]^+^ = 164.1, 146.1 (–H_2_O).

**(*E*)-2-(4-((*tert*-Butyldimethylsilyl)oxy)cyclohept-2-en-1-ylidene)propanenitrile (8) (See [12])**

To a solution of **7 **(220 mg, 1.30 mmol), imidazole (410 mg, 6.00 mmol), and DMAP (3.00 mg, 0.030 mmol) in CH_2_Cl_2_ at 0°C was added *tert*-butyldimethylsilyl chloride (TBSCl, 240 mg, 1.60 mmol). The reaction mixture was stirred at 0°C for 15 minutes and then warmed to r.t. for another 1.5 h. The reaction was quenched by the addition of water (25 mL). The organic phase was removed, and the aqueous phase was extracted with CH_2_Cl_2_ (3 × 15 mL). The combined organic phases were washed with brine (30 mL), dried over MgSO_4_, filtered, and concentrated *in vacuo*. The product was purified by column chromatography (40 g silica gel cartridge, gradient elution (0–5% EtOAc-hexanes); yield of **8**, 0.30 g (1.1 mmol, 80%).

*R_f_* = 0.36 (EtOAc-hexanes, 1 : 20).

^1^H NMR (600 MHz, CDCl_3_): *δ* = 6.23 (dd, 1H, *J *= 12.0, 2.0 Hz, H-5), 6.03 (dd, 1H, *J *= 12.0, 2.0 Hz, H-4), 4.49–4.43 (m, 1H, H-6), 2.82 (td, 1H, *J *= 14.0, 5.0 Hz, H-9), 2.52 (ddd, 1H, *J *= 14.0, 9.5, 5.0 Hz, H-9), 1.96–1.89 (m, 4H, H-7, C2-CH_3_), 1.84–1.70 (m, 3H, H-7, H-8), 0.90 (s, 9H, SiC(CH_3_)_3_), 0.10 (s, 3H, Si(CH_3_)_2_), 0.09 (s, 3H, Si(CH_3_)_2_).

^13^C NMR (150 MHz, CDCl_3_): *δ* = −4.8 (Si(CH_3_)_2_), −4.7 (Si(CH_3_)_2_), 16.2 (C2CH_3_), 18.1 (SiC(CH_3_)_3_), 22.2 (C8), 25.8 (SiC(CH_3_)_3_), 34.3 (C9), 35.8 (C7), 70.6 (C6), 105.2 (C3), 120.0 (C2), 125.0 (C5), 144.2 (C4), 152.9 (C1).

MS (APCI): *m/z* [M + H]^+^ = 277.9.

**(*E*)-2-(4-((*tert*-Butyldimethylsilyl)oxy)cyclohept-2-en-1-ylidene)propanal (9) (See [12])**

To a solution of **8 **(260 mg, 0.940 mmol) in ether (10 mL) at −78°C was added DIBAL-H (1 M in CH_2_Cl_2_, 1.41 mL, 1.40 mmol). The reaction mixture was stirred for 15 minutes at −78°C, then warmed to 0°C, and stirred for 2 h. The reaction was quenched by the addition of EtOAc (3 mL) at 0°C. It was then poured into a vigorously stirred slurry of silica gel (2.30 g, ~9 times the mass of the nitrile) and ether (15 mL) under argon at r.t. The slurry was stirred for 1 h. The silica gel was removed by vacuum filtration and rinsed with EtOAc (100 mL). The filtrate was concentrated *in vacuo*, yield of **9**, 0.25 g (0.91 mmol, 96%).

*R_f_* = 0.25 (EtOAc-hexanes, 1 : 20).

^1^H NMR (600 MHz, CDCl_3_): *δ* = 10.2 (s, 1H, H-1), 6.49 (d, 1H, *J *= 13.0 Hz, H-5), 6.08 (d, 1H, *J* = 13.0 Hz, H-4), 4.48 (m, 1H, H-6), 3.23 (td, *J *= 13.0, 5.0 Hz, 1H, H-9), 2.51–2.42 (m, 1H, H-9), 1.95–1.88 (m, 1H, H-7), 1.86–1.74 (m, 3H), 1.80 (s, 3H, C2-CH_3_), 0.93 (s, 9H, SiC(CH_3_)_3_), 0.12 (s, 3H, Si(CH_3_)_2_), 0.11 (s, 3H, Si(CH_3_)_2_).

^13^C NMR (150 MHz, CDCl_3_): *δ* = −4.8 (Si(CH_3_)_2_), −4.7 (Si(CH_3_)_2_), 10.9 (C_2_CH_3_), 18.1 (SiC(CH_3_)_3_), 23.8 (C8), 25.8 (SiC(CH_3_)_3_), 28.4 (C9), 35.5 (C7), 70.5 (C6), 129.9 (C3), 133.0 (C2), 143.6 (C5), 153.9 (C4), 191.0 (C1).

LRMS (EI): *m/z* (%) = 281.1 (2).

***tert*-Butyldimethyl(((*E*)-4-((*E*)-4-(2,6,6-trimethyl-cyclohex-1-en-1-yl)but-3-en-2-ylidene)cyclohept-2-en-1-yl)oxy)silane (11) (See [1, 12])**

To a solution of **10** (4.00 g, 8.40 mmol) in CH_2_Cl_2_ (30 mL) at r.t. in the dark was added potassium *tert*-butoxide (940 mg, 8.40 mmol) and 18-crown-6 (55.0 mg, 0.210 mmol). The solution was stirred for 15 minutes at r.t., then a solution of **9** (1.20 g, 4.20 mmol) in CH_2_Cl_2_ was added. The reaction mixture was stirred in the dark for 5 h at r.t. The reaction was quenched by the addition of water (100 mL) and extracted with ether (3 × 100 mL). The combined organic layers were washed with brine (100 mL), dried over Na_2_SO_4_, filtered, and concentrated *in vacuo*. The product was purified by column chromatography (330 g silica gel cartridge, gradient elution 0-1% EtOAc-hexanes); yield of **11**, 0.59 g (1.5 mmol, 35%).

*R_f_* = 0.9 (EtOAc-hexanes, 1 : 10).

^1^H NMR (400 MHz, CDCl_3_): *δ* = 6.52 (d, 1H, *J* = 16.0 Hz), 6.49 (dd, 1H, *J* = 12.0, 2.0 Hz), 6.20 (d, 1H, *J *= 16.0 Hz), 5.70 (dd, 1H, *J *= 12.0, 2.0 Hz), 4.53–4.46 (m, 1H), 2.71 (td, 1H, *J *= 13.5, 5.0 Hz), 2.31–2.20 (m, 1H), 2.10–1.94 (m, 2H), 1.90 (s, 3H), 1.87–1.76 (m, 2H), 1.74 (s, 3H), 1.72–1.54 (m, 4H), 1.47 (m, 2H), 1.06 (s, 3H), 1.05 (s, 3H) 0.92 (s, 9H), 0.12 (s, 3H), 0.11 (s, 3H).

MS (ES): *m/z* [M + H]^+^ = 400.1.

**(*E*)-4-((*E*)-4-(2,6,6-Trimethylcyclohex-1-en-1-yl)but-3-en-2-ylidene)cyclohept-2-enone (12) (See [1, 7, 12])**

To a solution of **11** (390 mg, 0.960 mmol) in THF (32 mL) in the dark, at 0°C, was added TBAF (1 M in THF, 1.90 mL, 1.92 mmol). The reaction mixture was stirred in the dark at 0°C for 5 h. The reaction was quenched by the addition of water (35 mL) and extracted with EtOAc (3 × 35 mL). The combined organic layers were washed with brine (50 mL), dried over Na_2_SO_4_, filtered, and concentrated *in vacuo*. The allylic alcohol (540 mg, yellow solid, *R_f_* = 0.24 (hexanes-EtOAc 10 : 1)) was dissolved in CH_2_Cl_2_ (15 mL) and cooled to −20°C in the dark. MnO_2_ (85% activated, 1.70 g, 20.0 mmol) was added, and the mixture was stirred for 10 minutes in the dark at −20°C. The reaction mixture was then warmed to r.t. and stirred for 2 h in the dark. The reaction mixture was then vacuum filtered through a pad of Celite, and the cake was washed with CH_2_Cl_2_ (5 × 10 mL). The bright yellow filtrate was concentrated *in vacuo*. The product was purified by column chromatography (40 g silica gel cartridge, gradient elution 0–20% EtOAc-hexanes); yield of **12**, 0.13 g (0.45 mmol, 47%).

*R_f_* = 0.18 (EtOAc-hexanes, 1 : 20).

^1^H NMR (400 MHz, CDCl_3_): *δ* ppm 7.39 (d, 1H, *J *= 12.0 Hz), 6.61 (d, 1H, *J *= 16.0 Hz), 6.45 (d, 1H, *J *= 16.0 Hz), 5.96 (d, 1H, *J *= 12.0 Hz), 2.65 (t, 2H, *J* = 6.5 Hz), 2.62 (t, 2H, *J* = 6.5 Hz), 2.06 (m, 5H), 1.96–1.87 (m, 2H), 1.76 (s, 3H), 1.70–1.62 (m, 2H), 1.57 (s, 1H), 1.51 (m, 2H), 1.07 (s, 6H).

MS (ES): *m/z* [M + H]^+^ = 285.1.

**(E)-2-((E)-4-((E)-4-(2,6,6-trimethylcyclohex-1-en-1-yl)but-3-en-2-ylidene)cyclohept-2-en-1-ylidene) Acetaldehyde (1a) (See [12])**

Into a dried round bottom flask containing NaH (60% dispersion in oil, 151 mg, 4.50 mmol) and THF (7 mL) was added diethyl cyanomethylphosphonate (0.710 mL, 4.50 mmol) over 15 minutes. The solution was stirred for 20 minutes at r.t., then the flask was wrapped in aluminum foil. A solution of the **12** (130 mg, 0.450 mmol) in THF (6 mL) was added to the reaction mixture, and the reaction was stirred for 3 h at r.t. in the dark. The reaction was poured into 30 mL of ice water and then extracted with ether (4 × 20 mL). The combined organic phases were washed with brine (20 mL), dried over MgSO_4_, filtered, and concentrated *in vacuo*. The intermediate nitrile was purified by column chromatography (40 g silica gel cartridge, isocratic 5% EtOAc-hexane); yield of pentaene nitrile 0.14 g (0.45 mmol, 99%).

*R_f_* = 0.36 (EtOAc-hexanes, 1 : 50).

To a solution of the nitrile (140 mg, 0.450 mmol) in Et_2_O (60 mL) at −78°C in the dark was added DIBAL-H (1 M in CH_2_Cl_2_, 3.00 mL, 3.00 mmol). The reaction mixture was stirred at −78°C for 10 minutes, then warmed to r.t., and stirred for 30 minutes in the dark. It was then poured into a vigorously stirred slurry of silica gel (5.50 g, ~40 times the mass of the nitrile) and ether (20 mL) under argon at r.t. The slurry was stirred for 30 minutes at r.t. The silica gel was removed by filtration under argon, and the silica gel was washed with ether (100 mL). The bright yellow filtrate was concentrated *in vacuo*, yield of *E/Z*-**1** 0.14 g (0.45 mmol, 96%, *E* : *Z* ratio ~1 : 1). The product was purified by HPLC using a normal phase semiprep column (Phenomenex Luna 5 *μ* silica 100 Å, 250 mm × 10 mm × 5 mm), isocratic elution 5% ether-hexanes, and flow rate of 10 mL min^−1^ and monitored at 350 and 210 nm. For optimal separation of isomers 1 mg aldehyde in 0.2 mL hexanes (HPLC grade) was injected per run. This provided **1a** with an isomeric purity of 96% by HPLC (AUC at 350 nm), and** 1b** with an isomeric purity of >98% by HPLC (AUC at 350 nm) (see Figure 3, panels **1a** and **1b** at *t*_0_).

*R_t_*  = 8.13 min; *R_f_* = 0.35 (EtOAc-hexanes, 1 : 20).

^1^H NMR (400 MHz, CDCl_3_): *δ* = 10.07 (d, 1H, *J *= 8.0 Hz, H-15), 6.95 (d, 1H, *J *= 11.5 Hz, H-11), 6.57 (d, 1H, *J *= 16.0 Hz, H-8), 6.38 (d, 1H, *J* = 16.0 Hz, H-7), 6.26 (d, 1H, *J *= 11.5 Hz, H-12), 5.97 (d, 1H, *J *= 8.0 Hz, H-14), 2.89 (t, 2H, *J *= 6.5 Hz, H-21), 2.60 (t, 2H, *J *= 6.5 Hz, H-20), 2.12–2.00 (m, 3H, C9-CH_3_), 1.94 (m, 2H, H-4), 1.76 (s, 3H, C5-CH_3_), 1.71–1.60 (m, 2H, H-3), 1.51 (m, 2H, H-2), 1.13–1.03 (s, 6H, 2xC1-CH_3_).

MS (ES): *m/z* [M + H]^+^ = 311.0.

**(*Z*)-2-((*E*)-4-((*E*)-4-(2,6,6-trimethylcyclohex-1-en-1-yl) but-3-en-2-ylidene)cyclohept-2-en-1-ylidene) Acetaldehyde (1b)**

*R_t_*  = 7.69 min; *R_f_* = 0.35 (EtOAc-hexanes, 1 : 20).

^1^H NMR (400 MHz, CD_2_Cl_2_): *δ* ppm 10.15 (d, 1H, *J* = 8.0 Hz, H-15), 7.07 (m, 2H, H-11, H-12), 6.61 (d, 1H, *J* = 15.5 Hz, H-8), 6.40 (d, 1H, *J* = 15.5 Hz, H-7), 5.80 (d, 1H, *J *= 8.0 Hz, H-14), 2.57 (t, 2H, *J* = 7.0 Hz, H-21), 2.47 (t, 2H, *J* = 7.0 Hz, H-20), 2.03 (m, 5H, H-4, C9-CH_3_), 1.95–1.85 (m, 2H, H-21), 1.76 (s, 3H, C5-CH_3_), 1.72–1.62 (m, 2H, H-3), 1.30 (m, 2H, H-2), 1.07 (s, 6H, 2xC1-CH_3_).

MS (ES): *m/z* [M + H]^+^ = 311.0.

## Figures and Tables

**Figure 1:**
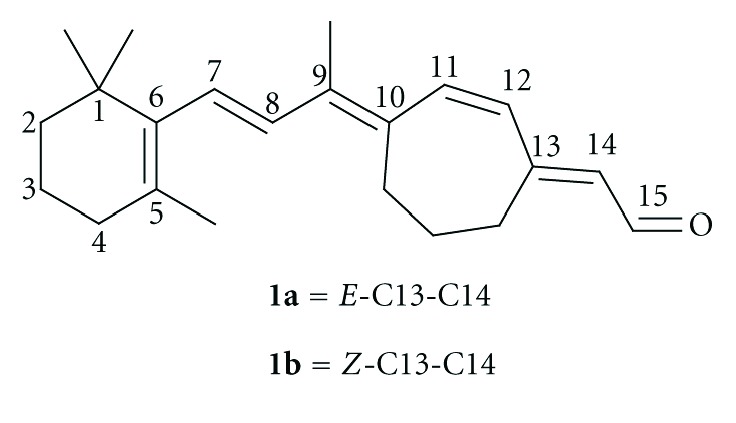
Ring-locked retinoid derivative of 11-*cis*-retinal.

**Scheme 1:**
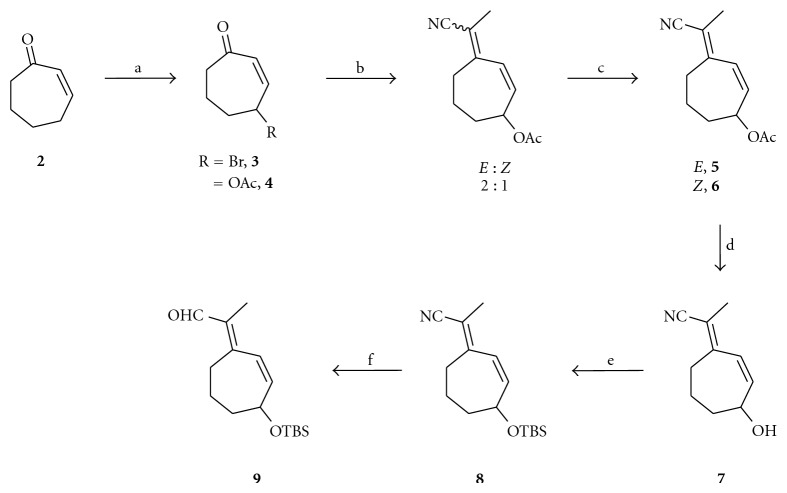
*Reagents and conditions*: (a) (i) NBS, ACHN, benzene, reflux, 2 h, and 34%; (ii) KOAc, TBAB, THF/H_2_O, r.t., 4 days, and 17%; (b) NaH, (EtO)_2_P(O)CH(CH_3_)CN, THF, 0°C to r.t., and 20 h; (c) isomer separation by SiO_2_ column chromatography, *E* 33%, and *Z* 17%; (d) K_2_CO_3_, MeOH, r.t., 1.5 h, and 99%; (e) TBSCl, imidazole, DMAP, CH_2_Cl_2_, r.t., 1 h, and 80%; (f) DIBAL-H, Et_2_O, −78°C to 0°C, 2 h, and 96%.

**Scheme 2:**
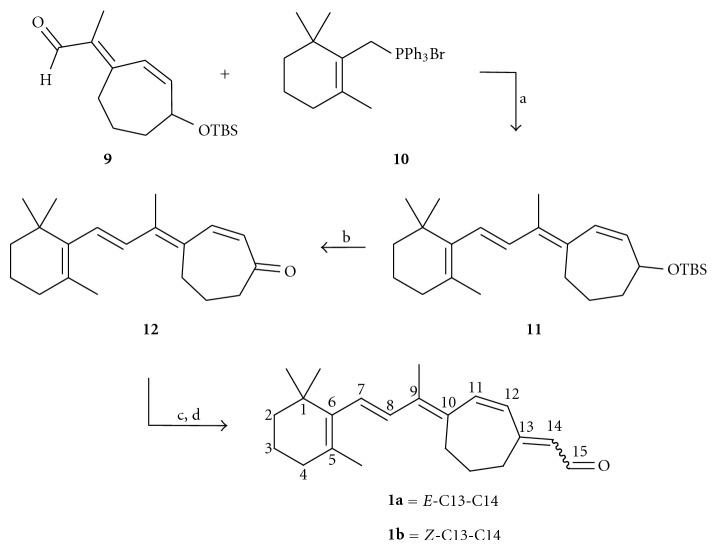
*Reagents and conditions*: (a) KO*t*Bu, 18-crown-6, CH_2_Cl_2_, r.t., 5 h, and 35%; (b) (i) TBAF, THF, 0°C, and 4.5 h; (ii) MnO_2_, CH_2_Cl_2_, −20°C to 0°C, 2 h, and 47%; (c) NaH, (EtO)_2_P(O)CH_2_CN, THF, r.t., 3 h, and 99%; (d) (i) DIBAL-H, Et_2_O, −78°C to r.t., and 30 min; (ii) SiO_2_ gel, ether, and *E*/*Z* 96%.

**Figure 2:**
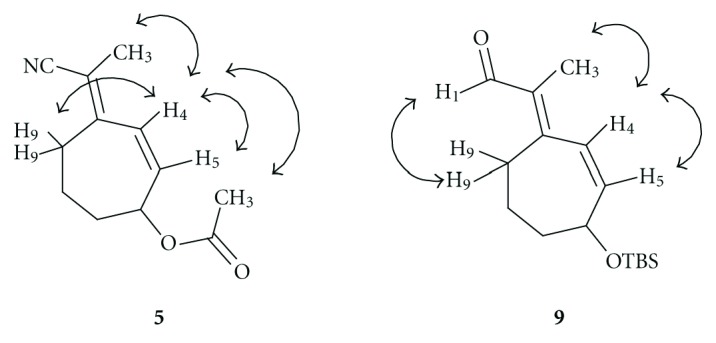
Key NOEs used to establish the geometry of the tetra-substituted double bond corresponding to the double bond between C9 and C10 of **1**.

**Figure 3:**
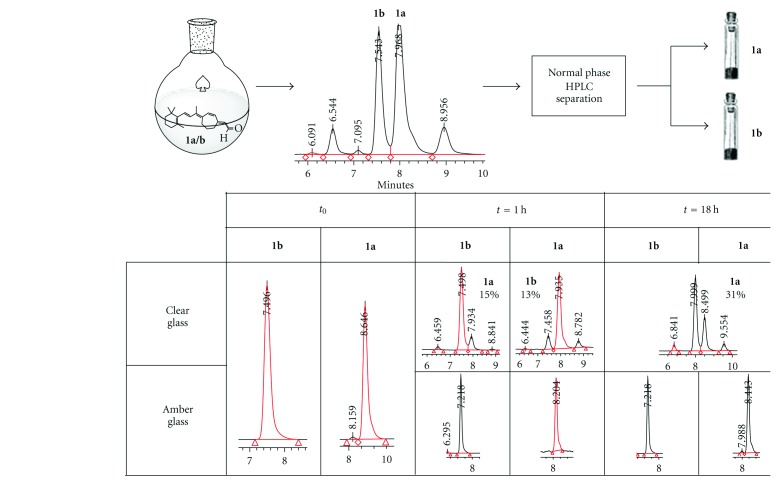
Isomerization between **1a** and **1b** under normal atmospheric conditions as monitored by HPLC (AUC at 310 nm). Note that samples stored in amber glass vial show no appreciable degradation or isomerization up to 18 h under argon at room temperature.

**Table 1:** Summary of the literature [1, 7] and experimental (exp) ^1^H NMR chemical shifts of key resonances (*δ* reported in ppm) for C13-C14 *E* and *Z*-isomers of **1**.

Proton	C13-C14 *Z*-isomer			C13-C14 *E*-isomer		
Reference	[1]	[7]	exp	[1]	[7]	exp
Solvent	CDCl_3_	CDCl_3_	CD_2_Cl_2_	CDCl_3_	CDCl_3_	CDCl_3_

H-7	6.32	—	6.40	6.32	—	6.38
H-8	6.52	—	6.61	6.52	—	6.57
H-11	7.02	7.00	7.07	6.92	—	6.95
H-12				6.22	6.21	6.26
H-14	5.79	5.78	5.80	5.93	5.93	5.97
H-15	10.12	10.11	10.15	10.03	10.03	10.07
